# Predictive Modeling Identifies Total Bleeds at 12-Weeks Postswitch to N8-GP Prophylaxis as a Predictor of Treatment Response

**DOI:** 10.1055/s-0041-1739514

**Published:** 2021-12-05

**Authors:** Pratima Chowdary, Kingsley Hampton, Victor Jiménez-Yuste, Guy Young, Soraya Benchikh el Fegoun, Aidan Cooper, Erik Scalfaro, Andreas Tiede

**Affiliations:** 1 Katharine Dormandy Haemophilia and Thrombosis Centre, Royal Free Hospital, London, United Kingdom; 2 Department of Cardiovascular Science, University of Sheffield, Sheffield, United Kingdom; 3 Department of Hematology, La Paz University Hospital-IdiPaz, Autónoma University, Madrid, Spain; 4 Hemostasis and Thrombosis Center, Cancer and Blood Disorders Institute, Children's Hospital Los Angeles, University of Southern California Keck School of Medicine, Los Angeles, California, United Sates; 5 Global Medical Affairs Biopharm, Novo Nordisk Health Care AG, Zürich, Switzerland; 6 Predictive Analytics, Real World Solutions, IQVIA, London, United Kingdom; 7 Real World Insights, IQVIA, Basel, Switzerland; 8 Department of Hematology, Hemostasis, Oncology and Stem Cell Transplantation, Hannover Medical School, Hanover, Germany

**Keywords:** factor VIII, hemophilia A, machine learning, predictive modeling, turoctocog alfa pegol

## Abstract

**Background** Predicting annualized bleeding rate (ABR) during factor VIII (FVIII) prophylaxis for severe hemophilia A (SHA) is important for long-term outcomes. This study used supervised machine learning-based predictive modeling to identify predictors of long-term ABR during prophylaxis with an extended half-life FVIII.

**Methods** Data were from 166 SHA patients who received N8-GP prophylaxis (50 IU/kg every 4 days) in the pathfinder 2 study. Predictive models were developed to identify variables associated with an ABR of ≤1 versus >1 during the trial's main phase (median follow-up of 469 days). Model performance was assessed using area under the receiver operator characteristic curve (AUROC). Pre-N8-GP prophylaxis models learned from data collected at baseline; post-N8-GP prophylaxis models learned from data collected up to 12-weeks postswitch to N8-GP, and predicted ABR at the end of the outcome period (final year of treatment in the main phase).

**Results** The predictive model using baseline variables had moderate performance (AUROC = 0.64) for predicting observed ABR. The most performant model used data collected at 12-weeks postswitch (AUROC = 0.79) with cumulative bleed count up to 12 weeks as the most informative variable, followed by baseline von Willebrand factor and mean FVIII at 30 minutes postdose. Univariate cumulative bleed count at 12 weeks performed equally well to the 12-weeks postswitch model (AUROC = 0.75). Pharmacokinetic measures were indicative, but not essential, to predict ABR.

**Conclusion** Cumulative bleed count up to 12-weeks postswitch was as informative as the 12-week post-switch predictive model for predicting long-term ABR, supporting alterations in prophylaxis based on treatment response.

## Introduction

Prophylaxis is considered the standard of care for the prevention and management of bleeding in patients with hemophilia A. 1 2 Extended half-life (EHL) recombinant factor VIII (rFVIII) molecules were developed to offer reduced dosing frequency and higher factor activity levels than standard half-life FVIII molecules. 1 Switching patients to prophylaxis with an EHL rFVIII molecule can provide meaningful improvements in health-related quality of life. 3 However, there is significant interpatient variability in response to rFVIII treatments, which may relate to the patient's age, body mass, bleeding phenotype, genotypic variation, ABO blood group, physical activity, and joint status, among other variables. 1 4 5 6

Identifying patient characteristics that predict long-term outcomes could help to inform clinical decisions for prophylaxis optimization. Annualized bleeding rate (ABR) is an established outcome measure of prophylaxis efficacy as it correlates with long-term joint destruction. 7 8 Previous attempts to predict and improve ABR have been focused on the use of FVIII pharmacokinetic (PK) parameters. Collins et al established that time per week spent at FVIII activity below 1 IU/dL was associated with increased total bleeds. 9 Valentino et al showed that peak FVIII levels, area under the curve, and time spent per week with FVIII levels >20 IU/dL were linked to bleeding risk. 4 More recently, Tiede et al demonstrated that bleeding risk can change over time and is influenced by factors independent to PK parameters, suggesting that other demographic and clinical characteristics are required to predict long-term treatment response. 10 Notably, the aforementioned approaches to predict ABR relate to evidence using standard half-life FVIII molecules for prophylaxis. Since then, guidelines have suggested to target higher trough levels, 2 which is achievable with EHL rFVIII molecules. It is reasonable to reassess predictors of long-term clinical response, as these may have changed with improvements in prophylaxis.

Predictive modeling involves the use of analytic techniques to predict clinical outcomes. Machine learning is a powerful computational approach used to recognize patterns in complex, multivariate datasets that include clinical variables and outcomes, enabling the development of predictive modeling. A significant advantage of machine learning techniques is their ability to handle highly variable datasets, collinearity, and missing data. Importantly, they can be used to identify how much a variable contributes to predicting a subsequent outcome. Rapid advances in machine learning techniques have enabled the application of predictive modeling to data from randomized controlled trials, showing significant promise in using patient and clinical characteristics to identify variables that are strongly associated with a chosen treatment outcome across a range of indications. 11 12 13

N8-GP is an EHL human rFVIII product that has the potential to provide a simplified prophylaxis treatment for patients with hemophilia A due to its fixed, body-weight-based dosing regimen (50 IU/kg every 4 days [Q4D]). 14 The mean trough level observed during prophylaxis with N8-GP (turoctocog alfa pegol; Esperoct, Novo Nordisk A/S, Bagsvaerd, Denmark) at 50 IU/kg every 4 days was 3 IU/dL. 15 The long-term safety and efficacy of N8-GP were demonstrated in the pivotal pathfinder 2 trial (NCT01480180), which is the only clinical trial of an EHL rFVIII molecule where most patients received a fixed dosing regimen for prophylaxis to date. In most other studies of EHL rFVIII molecules, patients received prophylaxis that was individualized by investigators, or patients were stratified to receive different regimens based on bleeding rate during a run-in phase. 16 17 For these studies, the application of machine learning techniques to identify predictive patterns is difficult, because the intensity of therapy was, at least to some extent, influenced by clinical outcomes. Due to its unique design, pathfinder 2 provides for the first time an opportunity to identify predictive patterns in the setting of prophylaxis with an EHL rFVIII molecule.

The purpose of this posthoc analysis was to develop a predictive model to identify pre- and post-N8-GP prophylaxis variables that can act as predictors of clinical response to fixed-dose prophylaxis by applying a machine learning framework to data from the pathfinder clinical trial program.

## Methods

### Research Objectives

The objectives of this exercise included the following: (1) to identify pre-N8-GP prophylaxis variables (including baseline characteristics) associated with an ABR of ≤1 or >1 bleed/year at the end of the pathfinder 2 main phase; (2) to identify post-N8-GP prophylaxis variables (including treatment-related variables, and patient characteristics) associated with an ABR of ≤1 or >1 bleed/year at the end of the pathfinder 2 main phase. For the purposes of this analysis, the ABR threshold of 1 (rather than “0”) was chosen as the outcome of interest, as the ability to identify and distinguish early bleeds varies between patients, and it is not uncommon for some patients to treat pain as an early bleed.

### Data Source

Data from the pathfinder trials (pathfinder 1, NCT01205724; pathfinder 2, NCT01480180; pathfinder 3, NCT01489111; pathfinder 5, NCT01731600; pathfinder 7, NCT02920398) were investigated for the application of supervised machine learning for predictive modeling. Of these, the pivotal pathfinder 2 trial 18 19 20 was selected for analysis due to the comprehensive array of variables from a sufficient sample size of patients. Pathfinder 2 was a phase III, open-label trial investigating long-term safety, PK, and efficacy of N8-GP used for prophylaxis (50 IU/kg Q4D) or on-demand treatment in 186 previously treated patients (aged ≥12 years) with severe hemophilia A. The full details of pathfinder 2 have been reported previously. 18 19 20

### Model Overview

Predictive modeling typically includes a predictive model learning phase, where data are collected for input into the model, and a predictive model outcome period, where the outcome of interest (ABR) is measured. The chosen clinical outcome of interest for prediction was ABR of ≤1 versus >1 at the end of the outcome period. Multiple predictive models using data from different time points in the study period were developed to identify variables pre- and post-N8-GP prophylaxis that were associated with patients achieving an ABR of ≤1 versus >1.

**Fig. 1:**
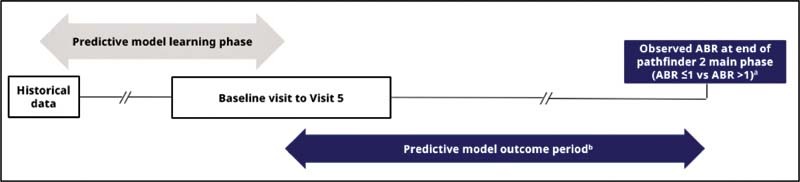
Predictive modeling methodology. ^a^ Multiple predictive models using data from different time points in the main phase were developed to identify which variables were associated with patients achieving an ABR of ≤1 versus >1 during the final stage of the outcome period. ^b^ ABR values were calculated for each model's outcome period, which varied between each predictive model. ABR, annualized bleeding rate.

Each predictive model learning phase used data collected from one of five separate time points in pathfinder 2. Pre-N8-GP prophylaxis models learned from data collected at screening visit ( *“baseline model”* ) or Visit 2 (prophylaxis initiation; *“baseline and PK model”* ). Post-N8-GP prophylaxis models learned from data collected between Visit 2 and 3 (i.e., up to 4 weeks postprophylaxis switch; *“post-N8-GP 4-week prophylaxis model”* ), Visit 4 (i.e., up to 8 weeks postprophylaxis switch; *“post-N8-GP 8-week prophylaxis model”* ), or Visit 5 (i.e., up to 12 weeks postprophylaxis switch; *“post-N8-GP 12-week prophylaxis model”* ), as well as the data collected at baseline ( Fig. 1 ). The 12-week post-N8-GP prophylaxis time window was chosen as the maximum duration of the predictive model learning phase as patients tend to be followed more closely during this period by clinicians, where initial treatment response becomes apparent, and this is often the earliest time that a change in treatment is considered.

Within each model cohort, patients were classified by observed clinical response at the end of the predictive model outcome period: ABR ≤1 versus >1. The predictive model outcome period was the time between the end of the learning phase of each model's time frame and the end of the pathfinder 2 main phase ( Fig. 1 ). As individual ABRs were not reported at the end of the main phase, ABR values were calculated based on the number of bleeds reported after each model's time point, and during the final 365 days of the predictive model outcome period prior to the last recorded site visit during the main phase for each patient. ABR values were calculated for each patient for each model's outcome period, which varied between each predictive model; patients received prophylaxis in the predictive model outcome period for approximately 15 months (median [min.–max. range]: 469 [107–747] days) between the baseline visit and end of the main phase, and approximately 13 months (median [min.–max. range]: 385 [100–654] days) of prophylaxis between the Visit 5 time point and end of the main phase. In patients where a complete 1-year outcome period was unavailable ( *n* = 44/166 [baseline model]; *n* = 70/161 [post-N8-GP 12-week prophylaxis model]), ABR was calculated by scaling the number of bleeds during the available predictive model outcome period. Patients on Q4D prophylaxis who switched to twice-weekly during the trial due to insufficient treatment response were included in the “ABR >1” group, irrespective of their ABR outcome. For patients who did not complete the main phase, ABR was calculated at the time of withdrawal.

Each predictive model included a cohort of patients from pathfinder 2 who received consistent N8-GP prophylaxis (50 IU/kg Q4D). Data were excluded if the patient received on-demand treatment or did not receive at least 90 days of prophylaxis exposure beyond the model's learning time point. As such, the number of patients in each model varied by time point, ranging from 166 patients in the pre-N8-GP prophylaxis models to 161 in the post-N8-GP 12-week prophylaxis model.

### Variable Selection

Standardized predictor variables for predictive modeling were selected from the range of patient and treatment characteristics collected at screening visit and after the switch to N8-GP prophylaxis. Variables that were expected to have clinical relevance were identified and reviewed by two clinicians before being selected for inclusion in the predictive analyses ( Supplementary Table S1 , available in the online version). Variable selection was based on clinical experience and availability of patient data; no additional criteria for variable selection were applied. Patient-reported outcomes such as EQ-5D were selected initially; however, preliminary model analyses did not support their retention for predictive modeling and hence these variables were excluded.

### Predictive Modeling Methodology

Two machine learning techniques were used for predictive modeling: penalized logistic regression and random forests (see the Supplementary Material , available in the online version). Additionally, interpretation methods for the tree-based models were used to elucidate the underlying drivers of treatment response. For explanation of predictions at the individual patient level, SHapley Additive exPlanations (SHAP) was used to quantify the contribution that each variable brought to the prediction made by the model. SHAP estimated how important each variable was by evaluating how well the model performed with and without that variable. 21 In this analysis, SHAP values greater than zero implied an increased association with an ABR ≤1; values less than zero implied an increased association with an ABR >1. Global ranking of how each variable contributed to the predicted clinical outcome at the group level was derived from mean absolute SHAP values.

### Model Validation

To validate the predictive potential of this approach, a repeated nested cross-validation strategy was implemented to alleviate “overfitting” and ensure the models could generalize well to new data. 22 In this, each patient could contribute to the learning (training set) phase of model development or act as a test patient (testing dataset or validation dataset), i.e., each patient was assigned a prediction from a corresponding predictive model. To facilitate interpretation, the association between variables and subsequent clinical outcome was described at the model level and individual patient level. A further description of model validation is provided in the Supplementary Material (available in the online version).

### Performance Metrics

The performance metrics of each predictive model were assessed using the area under the receiver operator curve (AUROC) to indicate how well variables collected at the model's time point could be used to predict the patients' observed ABR outcome at the end of the model outcome period. An AUROC of 1 indicated a perfect model and an AUROC of 0.5 indicated a model that performs equivalently to random chance. The predictive models were compared against a univariate benchmark to reference their performance. The univariate benchmark was historical ABR for pre-N8-GP prophylaxis models and total bleed count up to the model's time point for post-N8-GP prophylaxis models.

## Results

### Patient Characteristics

Of 175 patients initiated with N8-GP prophylaxis (50 IU/kg Q4D) in the pathfinder 2 main phase, a total of 166 received a consistent regimen and had sufficient follow-up for inclusion in the baseline model. Patient demographics and baseline characteristics for this cohort and the cohort of patients included in the post-N8-GP 12-week prophylaxis model are reported in Table 1 .

**Table 1:** Patient demographics and characteristics for the pathfinder 2 main phase cohort at screening visit (baseline) and Visit 5

	Screening visit (baseline)			Visit 5		
	Total ( *N* = 166) a	ABR ≤1 ( *n* = 87)	ABR > 1 ( *n* = 79)	Total ( *N* = 161)	ABR ≤1 ( *n* = 85)	ABR > 1 ( *n* = 76)
Age in years, mean (±SD)	30.5 (12.3)	31.3 (13.0)	29.7 (11.5)	30.5 (12.4)	31.0 (13.2)	29.8 (11.6)
Height in meters, mean (±SD)	1.76 (0.077)	1.77 (0.079)	1.74 (0.072)	1.76 (0.078)	1.77 (0.082)	1.74 (0.071)
BMI in kg/m ^2^ , mean (±SD)	24.4 (3.9)	24.4 (3.8)	24.4 (4.0)	24.4 (3.8)	24.5 (3.8)	24.4 (3.9)
Historical ABR, mean (±SD)	12.9 (27.7)	7.3 (12.2)	19.0 (37.2)	13.1 (28.1)	8.8 (15.2)	17.9 (36.9)
von Willebrand factor in IU/mL, mean (±SD)	1.00 (0.40)	1.09 (0.42)	0.90 (0.35)	1.00 (0.41)	1.08 (0.43)	0.91 (0.36)
Previous prophylaxis regimen, *n*	141	78	63	136	76	60
Previous on-demand regimen, *n*	25	9	16	25	9	16
Treatment history (months on previous prophylaxis/on-demand regimen), mean (±SD)	117.8 (108.7)	113.7 (106.6)	122.4 (111.4)	119.5 (109.7)	121.4 (114.1)	117.4 (105.4)

Abbreviations: ABR, annualized bleeding rate; BMI, body mass index; SD, standard deviation; vWF, von Willebrand factor.

a 14 of the 166 patients in this cohort switched to twice-weekly treatment and were classified in the “ABR > 1” group.

### ABR Outcome

Patients were classified by ABR at the end of the model outcome period (which ended at approximately 15 months after the model's time point). In the baseline model's cohort, 87 patients had an ABR ≤1, and 79 patients had an ABR >1. Details regarding the duration of the predictive model learning phase and outcome period are reported in Table 2 . The majority ( *n* = 122) of patients were exposed to prophylaxis for >1 year in the predictive model outcome period.

**Table 2:** Duration and variables of the predictive model learning phase and predictive model outcome period, and performance of the pre-N8-GP prophylaxis and post-N8-GP prophylaxis predictive models

Predictive model	Pre-N8-GP prophylaxis models		Post-N8-GP prophylaxis models		
	Baseline model	Baseline and PK model	Post-N8-GP 4-week prophylaxis model	Post-N8-GP 8-week prophylaxis model	Post-N8-GP 12-week prophylaxis model
Patient count, *n*	166	166	165	162	161
*Predictive model learning phase*					
Time points of the predictive model learning phase	Baseline	Baseline	Baseline and up to 4 weeks postswitch	Baseline and up to 8 weeks postswitch	Baseline and up to 12 weeks postswitch
Variables included for multivariable models	• 33 baseline variables	• 33 baseline variables • 2 PK variables (trough, FVIII at 30 minutes)	• 33 baseline variables • 2 PK variables: mean at Visit 3 (trough, FVIII at 30 minutes) • Cumulative bleed count up to Visit 3 (up to 4 weeks postprophylaxis switch)	• 33 baseline variables • 2 PK variables: mean at Visit 4 (trough, FVIII at 30 minutes) • Cumulative bleed count up to Visit 4 (up to 8 weeks postprophylaxis switch)	• 33 baseline variables • 2 PK variables: mean at Visit 5 (trough, FVIII at 30 minutes) • Cumulative bleed count up to Visit 5 (up to 12 weeks postprophylaxis switch)
* Predictive model outcome period*					
Duration of the predictive model outcome period, median (min.–max.) days	469 (107–747)	470 (107–747)	441 (94–714)	413 (115–686)	385 (100–654)
Patients with ABR ≤1 at the end of the outcome period, *n*	87	87	85	80	85
Patients with ABR >1 at the end of the outcome period, *n*	79	79	80	82	76
Model performance: single-variable benchmark					
AUROC for historical ABR	0.5821	0.5821	–	–	–
AUROC for cumulative bleed count until visit	–	–	0.6588	0.7059	0.7480
Model performance: multivariable models					
Penalized logistic regression, AUROC (95% CI)	0.636 (0.628–0.644)	0.672 (0.664–0.681)	0.689 (0.680–0.698)	0.707 (0.700–0.715)	0.724 (0.716–0.731)
Random forest, AUROC (95% CI)	0.608 (0.597–0.619)	0.622 (0.612–0.631)	0.690 (0.684–0.697)	0.758 (0.750–0.766)	0.785 (0.778–0.792)

Abbreviations: ABR, annualized bleeding rate; AUROC, area under the receiver operator curve; CI, confidence interval; FVIII, factor VIII; PK, pharmacokinetic.

Note: Performance evaluation of the predictive models was assessed using AUROC. AUROC values were rated as the following: AUROC of 0.5: model performs no better than random; AUROC 0.5 to <0.6: poor performance; AUROC 0.6 to <0.7: moderate performance; AUROC 0.7 to <0.8: good performance; AUROC 0.8 to <0.9: excellent performance; AUROC 0.9 to <1.0: outstanding performance; AUROC of 1.0: perfect performance; interpretation of AUROC values is subjective and influenced by clinical context. The single-variable benchmark for screening and Visit 2 is based on thresholding historical ABR reported at the screening visits. The benchmark for Visit 3 to Visit 5 is based on thresholding the count of bleed events observed since the screening visits.

### Performance of Predictive Models

The performance of the predictive models and single-variable benchmarks are reported in Table 2 . Univariate benchmark models were included to compare minimum performance against performance of the pre- and post-N8-GP prophylaxis models developed by supervised learning methods.

For the baseline model, an AUROC of 0.636 and 0.608 was reported for the penalized logistic regression and random forests, respectively, indicating “moderate” performance of this model for predicting the observed ABR outcome at the end of the outcome period. Model performance was improved in the baseline and PK model with the addition of PK measures, as indicated by higher AUROC values (0.672 and 0.622, respectively). Both pre-N8-GP prophylaxis models demonstrated improved performance compared with the single-variable benchmark (AUROC = 0.5821).

The post-N8-GP 12-week prophylaxis model was the most performant, with AUROC values of 0.724 and 0.785 for the penalized logistic regression and random forests, respectively, indicating “good” performance of this model for predicting the observed ABR outcomes. This model demonstrated marginally better performance to the single-variable benchmark of total bleed count until the model's time point (AUROC = 0.748). Model performance increased incrementally over time from the post-N8-GP 4-week prophylaxis model to the post-N8-GP 12-week prophylaxis model.

### Model Interpretability and SHAP Values

**Fig. 2:**
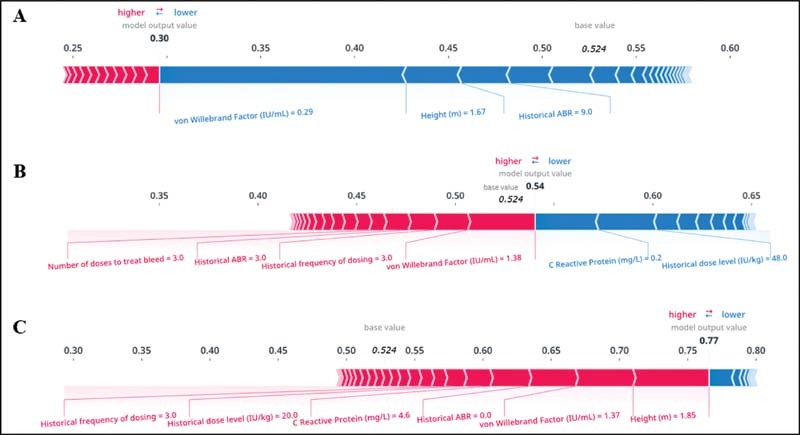
Individual predictions in the baseline model for ( **A** ) a patient with a strong negative outcome prediction, ( **B** ) a patient with an uncertain prediction, and ( **C** ) a patient with a strong positive outcome prediction. This plot reports local interpretability of individual patient predictions from three patient cases in the baseline model. The model outputs a score between 0 and 1. The “base value” of 0.524 is the percentage of patients with outcome ABR ≤1. The “model output value” for an individual patient is the sum of the base value and the SHAP value contributions of all individual variables. A model output value of less than the base value results in a prediction of ABR >1, and greater than the base value results in a prediction of ABR ≤1. Variables that increase the base value and predict ABR ≤1 are in *pink* , and visual size shows the magnitude of the effect. Variables that decrease the base value and predict ABR >1 are in *blue* . ABR, annualized bleeding rate; SHAP, Shapley Additive Explanations.

SHAP values were used to quantify the contribution of each variable to the prediction made by the model. Fig. 2 depicts individual predictions from three patient cases in the baseline model. The overall prediction for a patient (model output value) and the confidence in that prediction are related to the sum of the underlying contributions made by each of their variables. 23

### Predictive Variables in the pre-N8-GP Prophylaxis Models

**Fig. 3:**
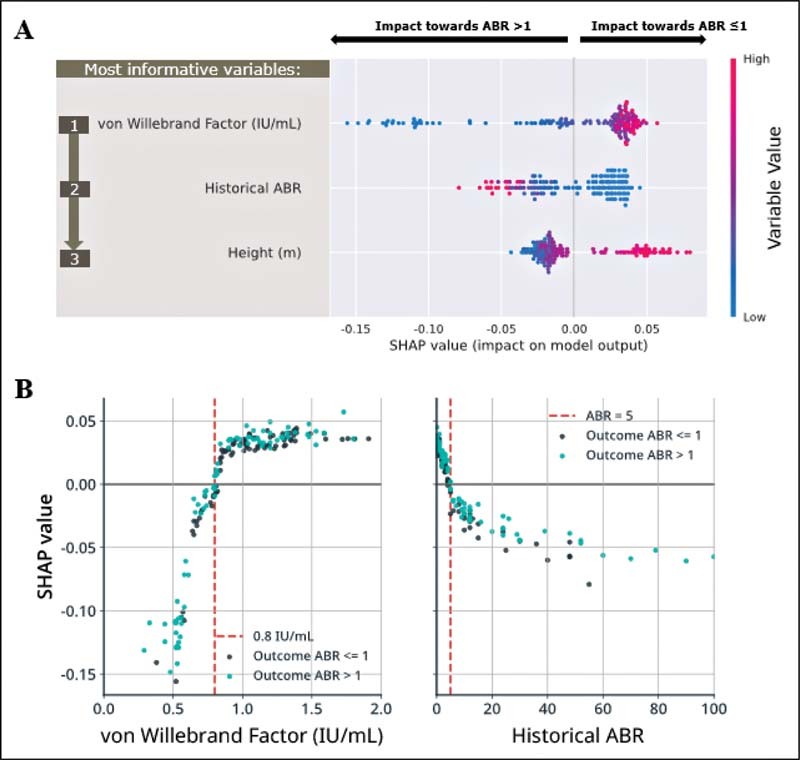
Global interpretability of ( **A** ) the baseline model and ( **B** ) the post-N8-GP 12-week prophylaxis model. Plot (A) reports variable importance and variable effect. The three highest performing clinically relevant variables in the baseline model are ranked according to mean absolute SHAP values, and shown as distributions across individual patients. Each dot indicates a single patient's SHAP value; the rank on the *y* -axis is determined by the mean absolute contribution of the variable to the model's output; the position on the *x* -axis indicates the SHAP value; the coloring indicates the value of the variable (e.g., high vWF level in *red* , low vWF level in *blue* ). Mean absolute SHAP values for global interpretation of variable importance in the baseline model are reported in Fig. 4 . Plot (B) reports SHAP values for each patient extracted from the random forest model using data collected at baseline/screening visit. Each dot indicates a single patient's SHAP value; coloring indicates ABR outcome during the end of the outcome period for each patient-level SHAP value. The *red line* indicates the variable threshold for positive and negative SHAP values. For both plots, instances with SHAP values greater than zero correspond to patients with variable values that influence the model toward predictions of ABR ≤1, whereas instances with SHAP values less than zero correspond to patients with variable values that influence the model toward predictions of ABR >1. ABR, annualized bleeding rate; SHAP, Shapley Additive Explanations; vWF, von Willebrand factor.

**Fig. 4:**
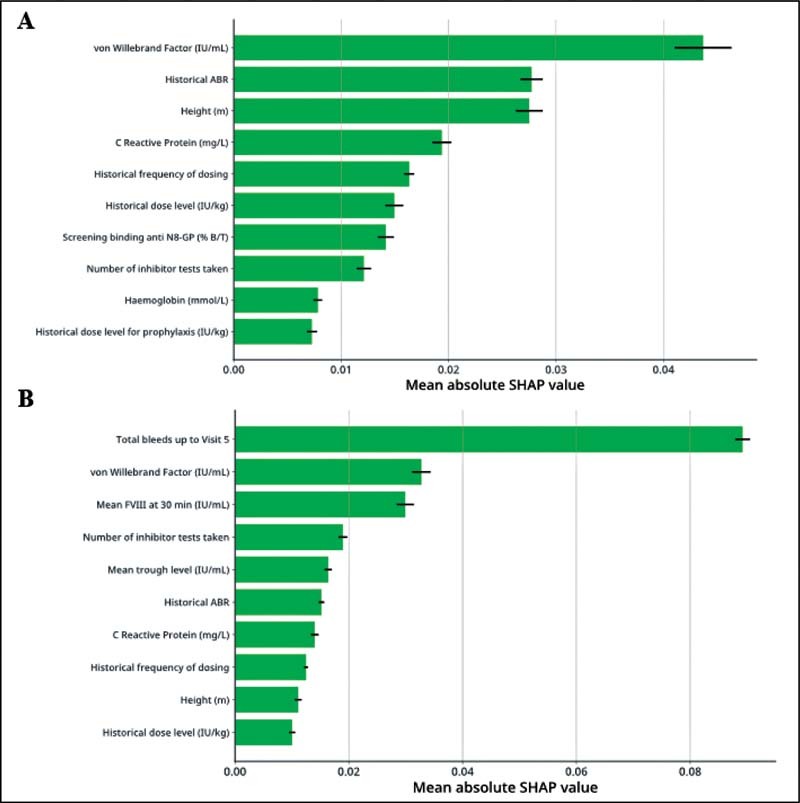
Mean absolute SHAP values for global interpretation of variable importance in the ( **A** ) baseline model and ( **B** ) the post-N8-GP 12-week prophylaxis model. The figure depicts mean absolute SHAP values for the 10 most informative variables in (A) the baseline model and (B) the post-N8-GP 12-week prophylaxis model for predicting observed ABR outcomes during the end of the outcome period. The SHAP value represents the amount a variable contributes to the model output. Error bars report standard error of the mean. ABR, annualized bleeding rate; SHAP, Shapley Additive Explanations.

The local interpretability for each predictor was grouped to interpret the overall effect of each variable on the model, which facilitates insight into associations, but not causality. When ranked by importance, SHAP analysis for global interpretability identified that the von Willebrand factor (vWF) level and historical ABR were the most impactful clinically relevant baseline variables for predicting observed ABR ( Fig. 3A ). Mean absolute SHAP values in Fig. 4A illustrate the global importance of the variable and Fig. 3A demonstrates the local explanation summary, demonstrating the direction of relationship between the variable and outcome. Baseline vWF had an asymmetric distribution of SHAP values, with extreme negative variable values influencing model predictions more than extreme positive values.

SHAP analysis of variables at a patient level identified that patients with a threshold vWF level <0.8 IU/mL or historical ABR >5 demonstrated negative SHAP values, indicating an increased association with an ABR >1. Patients with a higher vWF level >0.8 IU/mL or lower historical ABR <5 demonstrated positive SHAP values, indicating an increased association with an ABR ≤1 ( Fig. 3B ). An increase in the vWF level beyond 0.8 IU/mL was not associated with increasing odds of the patient having an ABR ≤1, as indicated by the plateauing SHAP values; however, decreasing levels of vWF below the 0.8 IU/mL threshold correlated with decreasing SHAP values, indicating a stronger association with patients reporting an ABR >1. The impact of height on predictions was most pronounced for the tallest patients (>1.8 m), who demonstrated positive SHAP values, indicating an increased association with an ABR ≤1.

### Predictive Variables in the Post-N8-GP Prophylaxis Models

**Fig. 5:**
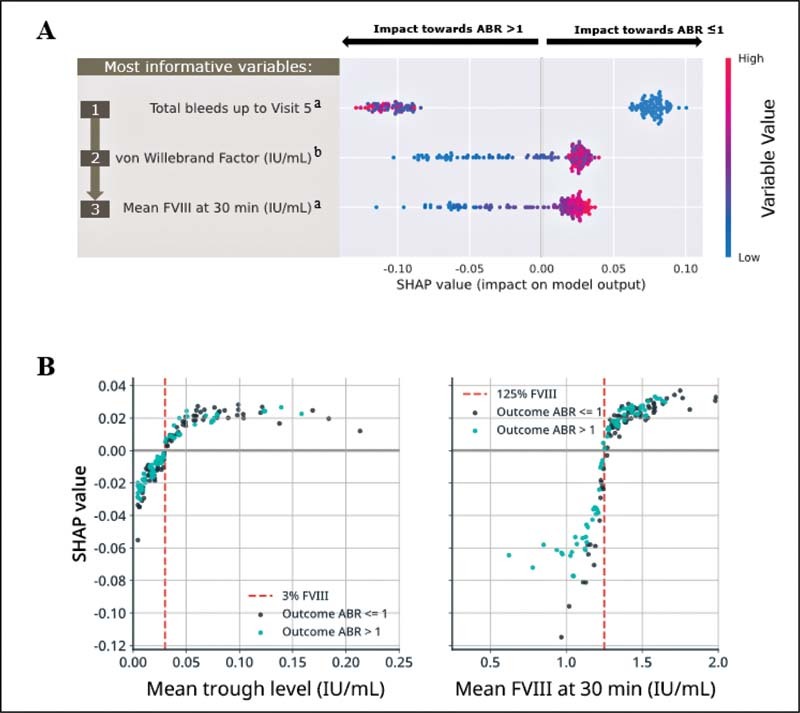
( **A** ) Global interpretability of the post-N8-GP 12-week prophylaxis model and ( **B** ) SHAP values for pharmacokinetic variables in the post-N8-GP 12-week prophylaxis model. ^a^ Post-N8-GP prophylaxis variables. ^b^ von Willebrand factor level at baseline (pre-N8-GP prophylaxis). Plot (A) reports variable importance and variable effect. The three highest performing clinically relevant variables in the post-N8-GP 12-week prophylaxis model are ranked according to mean absolute SHAP values, and shown as distributions across individual patients. Each dot indicates a single patient's SHAP value; the rank on the *y* -axis is determined by the mean absolute contribution of the variable to the model's output; the position on the *x* -axis indicates the SHAP value (positive values are predictive of lower ABR, and negative values are predictive of ABR >1); the coloring indicates the value of the variable (e.g., higher value in *red* , lower value in *blue* ). Mean absolute SHAP values for global interpretation of variable importance in the post-N8-GP 12-week prophylaxis model are reported in Fig. 4 . Plot (B) reports SHAP values for each patient extracted from the random forest model using data collected at 12 weeks post-N8-GP prophylaxis switch (for the pharmacokinetic variables). Each dot indicates a single patient's SHAP value; coloring indicates ABR outcome during the end of the outcome period for each patient-level SHAP value. The *red line* indicates the variable threshold for positive and negative SHAP values. For both plots, instances with SHAP values greater than zero correspond to patients with variable values that influence the model toward predictions of ABR ≤1, whereas instances with SHAP values less than zero correspond to patients with variable values that influence the model toward predictions of ABR >1. ABR, annualized bleeding rate; FVIII, factor VIII; SHAP, Shapley Additive Explanations.

Computation of SHAP values for global interpretability of the predictive model demonstrated that cumulative count of treated bleeds (i.e., “total bleed count”) up to 12 weeks post-prophylaxis switch, baseline vWF level, and mean FVIII at 30 minutes (Visit 5) were the most important clinically relevant variables for predicting observed ABR ( Fig. 5A ). Total bleed count up to 12 weeks postprophylaxis switch was the most informative variable for predicting observed ABR, and markedly more informative than baseline vWF level and mean FVIII at 30 minutes (Visit 5), as demonstrated by higher mean absolute SHAP values (0.089, 0.033, and 0.030, respectively; Fig. 4B ). The performance of cumulative bleed count up to 12 weeks was “good” (AUROC = 0.748) when assessed in isolation as a single-variable benchmark.

SHAP analysis for individual patient-level variable importance ranking identified that a higher mean trough level (>3%) and a higher FVIII at 30 minutes postdose (>125%) were associated with a patient achieving an ABR ≤1 ( Fig. 5B ). FVIII at 30 minutes postdose demonstrated a wider distribution of SHAP values compared with the trough level, indicating that FVIII at 30 minutes was more informative for predicting ABR in the model. At the patient level, SHAP analysis demonstrated that patients with a bleed count of zero universally had positive SHAP values (associated with a patient achieving an ABR ≤1), whereas patients with a bleed count greater than zero were universally shown to have negative SHAP values (i.e., encourage a prediction of ABR >1).

## Discussion

This posthoc analysis applied supervised machine learning techniques to data from pathfinder 2 to develop predictive models to identify which pre- and post-N8-GP prophylaxis variables act as predictors of clinical response to fixed-dose N8-GP prophylaxis. The most performant predictive model was the post-N8-GP 12-week prophylaxis model, which demonstrated that total bleed count during the initial 12-week period postswitch to N8-GP prophylaxis was the most informative variable in the model for predicting ABR at the end of the outcome period (which ended a median of approximately 13 months after the model's time point, i.e., long-term response to N8-GP prophylaxis), followed by baseline vWF level and mean FVIII at 30 minutes postdose. When assessed as a univariate benchmark, total bleed count up to 12 weeks was as performant as the post-N8-GP 12-week prophylaxis model, demonstrating the importance of clinical observation for predicting long-term outcomes.

The performance of the baseline model was “moderate” (AUROC = 0.636), and only marginally improved compared with the single-variable benchmark (historical ABR; AUROC = 0.5821). The model indicated that the vWF level was the most impactful baseline variable for predicting observed ABR. Patients who responded well to prophylaxis were those with higher baseline vWF levels (>0.8 IU/mL), which is consistent with previous studies demonstrating that higher vWF levels correlate with longer FVIII half-life and higher FVIII activity between doses that may subsequently lead to a reduced ABR. 16 24 The model identified a linear relationship between low vWF and higher bleeds, which plateaued beyond 0.8 IU/mL. The models identified that patients with greater height (>1.8 m) reported positive SHAP values that were associated with an ABR ≤1. Although taller patients may report lower body mass index (BMI) values, which is associated with reduced clearance, 25 BMI and body weight were included as variables in the analysis and not found to be informative predictors of ABR. Taller patients in this cohort may instead represent a group of patients who were possibly more athletic, or had different levels of activity; the association between a taller height and lower ABR is currently unclear. Overall, the “moderate” performance (AUROC = 0.636) of the baseline model indicated that baseline variables were limited in their performance to accurately predict ABR; as such, the association between a taller height and lower ABR may be a spurious finding. The limited performance of the baseline model confirms the dominant influence of the prophylaxis regimen on ABR outcomes.

The post-N8-GP 12-week prophylaxis model was the most performant and assessed by AUROC as “good” (AUROC = 0.785). The “good” (AUROC = 0.785) performance of this model indicates that the 12-week observation window (learning phase) postswitch to N8-GP prophylaxis is a sufficient period of time to monitor patients and determine whether a change in treatment or regimen may be required, or investigations for other causative factors. The post-N8-GP 12-week prophylaxis model included total bleed count; as a single-variable benchmark, total bleed count up to 12 weeks was more performant than all variables included in the baseline model (AUROC of 0.75 vs. 0.64, respectively). The post-N8-GP 12-week prophylaxis model and total bleed count univariate benchmark model demonstrate the importance of bleeding phenotype-adjusted tailoring of prophylaxis over baseline characteristics for predicting long-term ABR.

Previous studies investigating predictors of ABR identified that endogenous factor levels, adherence, BMI, primary dosing regimen, inhibitor development, presence of arthropathy, and intensity of physical activity as variables that can influence bleeding rates. 1 4 26 27 28 29 30 In the present analysis, the post-N8-GP 12-week prophylaxis model demonstrated that total bleed count up to 12 weeks postprophylaxis switch, baseline vWF level, and mean FVIII at 30 minutes postdose were the most important variables for predicting observed ABR with N8-GP prophylaxis. The mean absolute SHAP values demonstrated that the total bleed count was substantially more informative than all other baseline characteristics (vWF level) and PK measures (mean FVIII at 30 minutes and mean trough level), demonstrating the importance of clinical observation for predicting long-term outcomes. The “good” performance (AUROC = 0.748) of cumulative bleed count as a univariate benchmark model is in line with clinical observations, whereby patients who bleed less in the initial prophylaxis period of a trial are more likely to have fewer bleeds later in the trial. These results suggest that bleeding events following prophylaxis initiation are indicative of long-term outcomes and should prompt a review of the prophylactic regimen or investigation for local pathology that may be contributing to an excess of bleeds. In routine clinical practice, while prophylaxis tends to be adjusted based on treatment response, there is no agreed period for review or an agreed cut-off for number of bleeds before deciding on a treatment change. For the first time, this posthoc analysis confirms that a 12-week treatment period is adequate to initiate treatment review in the event of bleeds, for intensification of prophylaxis or investigation into other contributing factors.

The post-N8-GP 12-week prophylaxis model recognized that a higher trough level (>3%) and FVIII at 30 minutes (representing peak FVIII activity of N8-GP 15 ; >125%) were both associated with an ABR ≤1. Similarly, the literature reports that maintaining a higher FVIII trough level and achieving a higher FVIII peak provides increased protection from joint and nonjoint bleeding, and subsequent improvements in ABR. 4 10 30 FVIII at 30 minutes and trough level may act as surrogate markers for N8-GP PK area under the curve and decay of the curve. SHAP analysis indicated that the FVIII level at 30 minutes postdose was more informative than the trough level for predicting observed ABR. This result suggests that achieving the observed peak threshold of >125% is associated more strongly with a higher PK area under the curve and an ABR ≤1 than achieving a trough level >3%. However, this result may also reflect the quality of data collected, whereby values for FVIII level at 30 minutes are less anomalous than the trough level, and may therefore act as a more informative measure.

Previous investigations into predicting ABR outcomes using standard half-life FVIII focused on the association of FVIII PK measurements with ABR. 4 9 In this analysis with an EHL FVIII molecule, the inclusion of PK measurements improved model performance, as demonstrated by higher AUROC values for the baseline and PK model. However, the contribution of mean FVIII at 30 minutes and mean trough level to the predictive power of the post-N8-GP 12-week prophylaxis model was substantially lower than the total bleed count, as demonstrated by lower mean SHAP values (0.030 and 0.016 vs. 0.089, respectively). Although PK data may be used to inform decisions and individualize treatment in clinical practice (e.g., dose adjustments), 31 the results reported in the present analysis suggest that using clinical observations of total bleed count alone during the initial 12-week prophylaxis period (univariate assessment) in the context of fixed-dose prophylaxis with a high trough level is as good and potentially more informative to predict long-term prophylaxis response. The results confirm the value of a simplified, fixed-dose prophylaxis regimen for patients, with possible adaptation of treatment according to bleed count in the initial weeks postinitiation, rather than based on the residual clotting factor activity levels. 32

Limitations of this analysis included the modest sample size of patients ( *n* = 166), which was subject to selection bias, whereby patients included were those who remained in the study for longer and could have been more likely to be compliant or be motivated to remain in the study due to observed improvement. The analysis was based on main phase data from pathfinder 2 only; the performance of the predictive models may benefit from a longer observation time with refined outcomes of interest. Data were not available for joint status, which is considered a clinically relevant variable for phenotypic assessment prior to treatment initiation in hemophilia A patients. 33 Data included in the analyses were for patients receiving N8-GP prophylaxis Q4D, and hence there was limited scope to compare findings against other regimens, doses, or treatments. Due to the modest sample size of patients, validation was performed using nested cross-validation (i.e., “internal validation”). Although nested cross-validation is an acceptable method for validating a predictive model, the use of a separate dataset for external validation is more robust for confirming model performance. The dataset included anomalies in trough level data, and a proportion of trough level values was below the detection limit threshold (0.045 IU/mL). FVIII at 30 minutes and FVIII trough level were the only PK parameters from pathfinder 2 for which there were sufficient data for inclusion in the predictive models. Machine learning for predictive modeling was applied to an EHL PEGylated FVIII molecule; although the association between clinically relevant variables and predicted ABR outcomes is unlikely to be specific to N8-GP, results cannot be readily extrapolated to standard half-life FVIII molecules, or those with different half-life extension technology.

To refine the performance and value of the predictive models in future analyses, results should be replicated in data from other hemophilia clinical trials, as well as the real-world setting where patient behavior may be more variable, and where a broader set of variables may be available. As the performance of each predictive model improved with the inclusion of more variables, the availability of additional clinically relevant variables such as joint status, physical activity, and intensity of previous prophylaxis regimen may help to further increase model performance. Additionally, the availability and inclusion of other PK parameters, such as area under the curve, should be applied to future machine learning analyses to further investigate the role of PK. The ability to predict ABR as a continuous value or range of values (rather than as a binary outcome of ABR ≤1 vs. ABR >1) may affect the model, as the occurrence of a single bleed event would have a large impact on the defined outcome.

## Conclusion

Machine learning for predictive modeling is a novel approach to analyzing data from hemophilia clinical trials. In this posthoc analysis, applying supervised machine learning techniques for predictive modeling to data from the pathfinder 2 trial demonstrated that cumulative bleed count in the 12 weeks postprophylaxis switch was the most informative variable for predicting observed ABR at the end of the outcome period (approximately 13 months later), and probably more informative than PK assessments and baseline characteristics. This outcome confirms observations in clinical practice in which patients who bleed less in the initial period posttreatment switch are more likely to have fewer long-term bleeding events. Additionally, it places greater emphasis on phenotype-adjusted tailoring of prophylaxis according to bleed response, as well as investigations for other mechanisms of bleeding. The results confirm the value of simplified fixed-dose prophylaxis with adaptations based on both clinical outcomes and PK parameters. Furthermore, this analysis reports the first use of machine learning techniques applied to data from a hemophilia clinical trial, with the aim to identify results that can be applied in the clinic. The analysis should be highlighted as an example for the use of artificial intelligence techniques applied to data from future clinical trials in hemophilia for any therapeutic modality.

## Notes

**What is known about this topic?**

- The most recent iteration of the World Federation of Hemophilia guidelines 2 recommends a higher trough level for prophylaxis, which can be achieved using extended half-life (EHL) factor VIII (FVIII) molecules, and the use of personalized prophylaxis plans.
- Previous studies have attempted to predict bleeding risk and annualized bleeding rate (ABR) in people with hemophilia A utilizing FVIII pharmacokinetic (PK) parameters, and reported an association between bleeding risk and peak FVIII level, area under the curve, and time per week with FVIII activity below 1 IU/dL.
- However, bleeding risk can change over time and may be influenced by factors independent to PK parameters, suggesting that other demographic and clinical characteristics are required to predict long-term treatment response to prophylaxis.

**What does this paper add?**

- To our knowledge, this is the first analysis reporting the application of supervised machine learning techniques for predictive modeling to data from a hemophilia clinical trial (namely the pathfinder 2 trial in the setting of prophylaxis with the EHL recombinant FVIII molecule N8-GP).
- Predictive modeling in the present analysis identified that cumulative bleed count up to 12 weeks postswitch to N8-GP prophylaxis, baseline von Willebrand factor, and mean FVIII at 30 minutes postdose were the most informative variables for predicting ABR outcomes during the final year of the main phase (median of 385 days after the model's time point) in patients who received fixed-dose prophylaxis with N8-GP.
- Bleed count at 12 weeks post-N8-GP prophylaxis switch was more informative than PK measures (trough level, mean FVIII at 30 minutes postdose) and baseline characteristics for predicting long-term ABR, emphasizing the importance of bleeding phenotype-adjusted tailoring of prophylaxis.
