# Supplementary material for: Predictive Modeling Identifies Total Bleeds at 12-Weeks Postswitch to N8-GP Prophylaxis as a Predictor of Treatment Response
Source: Thromb Haemost. 2021 Dec 5;122(6):913–25. doi: 10.1055/s-0041-1739514 (PMC9251711; doi:10.1055/s-0041-1739514)
Supplement: Supplementary file 1 — Supplementary Material [file 10-1055-s-0041-1739514-s210227.pdf]

Supplementary Material

Methodology

Predictive Modeling Methods

Penalized logistic regression builds on a classical logistic regression approach by incorporating a penalty on the regression coefficients, which helps to alleviate overfitting; in particular, the L2 (ridge) penalty was implemented. The extent of influence of the penalty was a hyperparameter that was tuned during the learning phase. Random forests have several properties that are well suited to this use-case and are also, in some cases, complementary to logistic regression. Specifically, random forest is an ensemble of decision trees that are sensitive to interactions and nonlinear relationships between variables, as well as being robust to overfitting due to the combination of multiple decision trees on different subsamples of data in terms of patient observations and variables. Several hyperparameters can be tuned as part of the random forest algorithm, including the maximum depth of each decision tree and the minimum number of samples that must be present at each leaf node.

Model Validation Data Strategy

To validate model performance, it is generally preferred to use a subset of patients that are excluded from model learning (training set) and used exclusively for assessing the performance of the final model. Given the model sample size available here (in terms of unique patient counts), models were developed and evaluated using a nested cross-validation methodology, composed of eight inner folds and eight outer folds. The inner cross-validation was used to select optimal model hyperparameters via a grid search, which was then applied and evaluated in the outer cross-validation to measure performance on “unseen” data. Model performance was measured as the average performance across the outer test folds. To assess the stability of model performance, the average scores and confidence intervals were reported over 25 distinct runs of the nested cross-validation strategy. This combined approach of repeated nested cross-validation yields stable scores with minimal bias, while maximizing usage of the limited underlying dataset.

 © 2021. The Author(s).

Supplementary Table S1 Clinically relevant variables used for the predictive analyses

Variable types	Variable descriptions	Model
Patient demographics	Age (years)<br>Body mass index at baseline (kg/m^2)<br>Type of treatment regimen prior to trial (prophylaxis/on-demand)<br>Height at baseline (m)<br>Historical annualized bleeding rate<br>Body weight at baseline (kg)	Pre-N8-GP prophylaxis and post-N8-GP prophylaxis
History analysis	Frequency of dosing (interval in days)<br>FVIII product given (recombinant/plasma-derived)<br>Treated bleeds prior to prophylaxis (*n*)<br>Common doses used to treat a bleed (*n*)<br>Dose for on-demand treatment (IU/kg)<br>Time on previous regimen (months)<br>Dose for prophylaxis (IU/kg)<br>Mutation type [V1/V3] (mutation type)<br>Underlying gene defect (known/unknown)<br>History of hemophilia A among relatives (true/false)<br>Number of inhibitor tests (*n*)<br>Number of recovery tests (*n*)<br>Relatives with hemophilia A and inhibitors (true/false)	
Laboratory results analysis	Anti-PEG antibodies (titer)<br>Screening binding anti N8-GP (% B/T)<br>C-reactive protein (mg/L)<br>von Willebrand factor (IU/mL)<br>Hepatitis C antibodies (present/absent)<br>Hemoglobin (g/L)<br>N8-GP binding antibodies (titer)<br>Platelets (10^9/L)	
Physical examination analysis	Musculoskeletal system [at V1 screening] (score: 1 = normal; 2 = abnormal, not clinically significant; 3 = abnormal, clinically significant)<br>Skin [at V1 screening] (score: 1 = normal; 2 = abnormal, not clinically significant; 3 = abnormal, clinically significant)	
Vital signs analysis	Diastolic blood pressure (mm Hg)<br>Systolic blood pressure (mm Hg)	
Medical history	*Body system or organ class grouped as:*<br>• Musculoskeletal (*n* of events)<br>• Surgical and medical procedures (*n* of events)	
Pharmacokinetic	Mean FVIII trough level up until each model time point (IU/mL)<br>Mean FVIII activity at 30 minutes up until each model time point (IU/mL)	Pre-N8-GP prophylaxis (Visit 2)[a] and post-N8-GP prophylaxis
Bleeds	Cumulative number of treated bleeds up until each model time point (n)	Post-N8-GP prophylaxis

Abbreviations: FVIII, factor VIII; V, visit.
[a]The baseline and PK model included single measurements of trough level and FVIII activity at 30 minutes per patient.

 © 2021. The Author(s).
